# Graphene as a nanofiller for enhancing the tribological properties and thermal conductivity of base grease†

**DOI:** 10.1039/c9ra09201c

**Published:** 2019-12-20

**Authors:** Hui Fu, Guoping Yan, Meng Li, Hao Wang, Yapeng Chen, Chao Yan, Cheng-Te Lin, Nan Jiang, Jinhong Yu

**Affiliations:** College of Materials Science and Engineering, Wuhan Institute of Technology Wuhan 430073 Hubei China guopyan2006@163.com; Key Laboratory of Marine Materials and Related Technologies, Zhejiang Key Laboratory of Marine Materials and Protective Technologies, Ningbo Institute of Materials Technology and Engineering, Chinese Academy of Sciences Ningbo 315201 China jiangnan@nimte.ac.cn yujinhong@nimte.ac.cn; Center of Materials Science and Optoelectronics Engineering, University of Chinese Academy of Sciences Beijing 100049 China; School of Materials Science and Engineering, Jiangsu University of Science and Technology Zhenjiang 212003 China

## Abstract

During mechanical processes, violent friction and wear between the friction contact surfaces not only causes wear to mechanical components, reducing the instrument life, but also causes friction heat, reducing the working efficiency of machines during operation. The addition of graphene-reinforced grease to the mechanical friction surface can effectively reduce the friction coefficient and improve the thermal conductivity. In this work, the tribological properties and thermal conductivity of base grease with graphene were investigated systematically. The tribological results showed that the grease with 2 wt% graphene had the best tribological properties among all these greases. The wear scar diameter and average friction coefficient of graphene grease with 2 wt% graphene reached 0.43 mm and 0.10 (the values for base grease are 0.50 mm and 0.118), respectively. In addition, the average friction coefficient and wear scar diameter increased proportionally with the increasing load and frequency. The thermal conductivity of the grease with 4 wt% graphene reached 0.28 W (m K)^−1^, an increase of 55.5% in comparison with the base grease. It is proposed that the addition of graphene into the base grease effectively enhanced the tribological properties and thermal conductivity.

## Abstract 2

The addition of graphene-reinforced grease to the mechanical friction surface can effectively reduce the friction coefficient and accelerate heat transportation.

## Graphical abstract

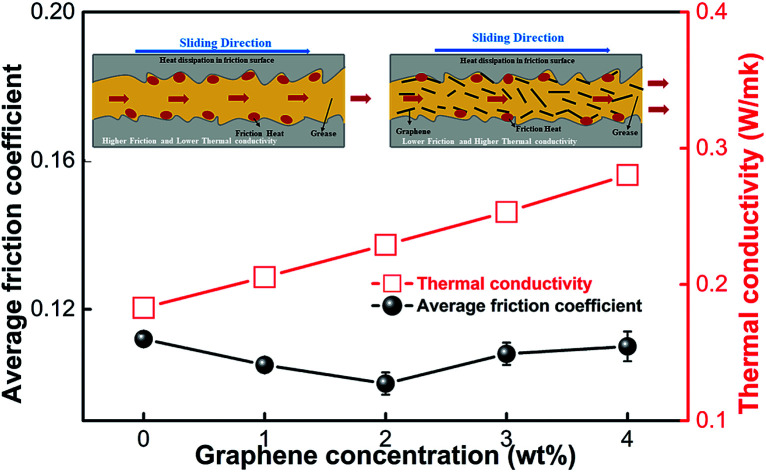

## Introduction

Increasing industrialization has provided peace and well-being, but it causes much energy waste. A portion of this energy waste is produced by friction and wear in mechanical systems. Many researchers have tried to control friction and wear by lubricating rubbing surfaces; one of the most effective ways is adding grease or oil to lubricate the surface to reduce the friction coefficient, which not only effectively decreases energy waste and improves the working efficiency of the machine, but also reduces damage to the equipment. Both grease and lubricating oil are superior lubricating materials.^1,2^ When they are added to gears or other mechanical friction surfaces, the friction coefficient and wear scar diameter (WSD) in particular obviously decrease. Grease and lubricating oil with some nanoparticles can ameliorate the tribological performance more effectively compared with grease and lubricating oil without nanoparticles.^3^ However, there is a disadvantage in that the nanoparticles gather together and settle down, as time goes by, when the nanoparticles are dispersed in oil. Compared with oil, there is no trouble in grease. Owing to its high viscosity, grease with nanoparticles retains its effectiveness. It can effectively enhance the anti-wear properties and decrease friction on metal–metal contact surfaces. As a lubrication additive dispersed in grease, many nanoparticles are available, such as inorganic nanoparticles, carbon nanoparticles and other 2D structural materials.^4–6^

Graphene, with a carbon atom possessing sp^2^ hybrid orbitals, is a carbon material with a two-dimensional structure, which has attracted lots of attention owing to its exceptional electric, thermal, physical and mechanical properties.^7^ Graphene can be validly applied in various fields, such as solar cells, supercapacitors, sensors, thermal conductivity composites and tribological materials.^8–12^ When adding graphene into lubricating materials, such as grease, the lubricity of the materials can be enhanced effectively.^13^ Besides, compared with the base lubricating material, the thermal conductivity of the composite is also improved.^14^

As an additive, graphene can validly improve the anti-wear and reduce the friction lubrication performance of grease, owing to its favourable mechanical properties and extremely smooth atomic surface. As a result of graphene's extremely small particle size and large surface area, the oxidation resistance of grease with graphene is greatly enhanced^15^. The use of graphene as an additive in base grease to enhance the tribology had been widely reported. Zhang^16^ added graphene prepared by physical stripping method to lithium-based grease, and studied the tribological properties and critical load pressures for grease with different graphene contents (0–2 wt%). Wu^17^ studied a composite nanomaterial composed of a bubble structure composed of molybdenum disulphide/redox graphene (MoS_2_/RGO) and found that adding it to grease greatly improved its wear resistance. Singh^18^ compared the tribological and rheological properties of grease with different nanoparticles added (redox graphene, calcium carbonate, α-alumina). Wang^19^ added graphene–copper nanoparticles (GN/Cu) prepared by reduction to grease, then studied the tribological properties of the GN/Cu grease. Kamel^20^ studied the effects of different concentrations of graphene on the tribological properties of calcium-based greases.

Graphene has been reported to improve the tribological properties and thermal conductivity of grease or composites in many relevant recently published studies. Actually, many researchers have found that graphene has excellent tribological properties and thermal conduction performance and they are now paying more attention to single fields, like tribological properties or thermal conductivity. However, these researchers have not combined the tribological properties with the thermal conductivity of grease.^21–23^ The relevant interpretation and wear mechanisms of graphene have been studied and the relevant published work can be used for ref. 24–27. Thus, we studied the performance of grease in terms of both the tribological properties and the thermal conductivity. We studied the performance of grease systematically, owing to the thermal properties being considered during the machine operational time.

In this work, the graphene dispersion in grease could enhance the tribological properties and thermal transportation. Graphene could be completely dispersed in grease owing to the “shear-thinning” phenomena. The friction and wear resistance were measured *via* the four-ball test. We also investigated the load-carrying and frequency-carrying capacity of graphene-reinforced grease to complete the tribological properties. The critical value of oil film rupture and the complete failure of oil were also studied carefully. At the same time, the thermal transportation properties of graphene-reinforced grease were studied and verified by infrared thermography.

## Experimental

### Materials

Graphene was purchased from Ningbo Morsh Tech. Co., Ltd. (China). The base grease was purchased from Shaoxing G&N Lubricate Materials Co., Ltd. (China).

### Preparation of graphene grease

Each graphene grease sample was prepared by adding graphene into the base grease at 25 °C and then mixing using a mixing machine (Flacktek SpeedMixer DAC 150 FVZ-K) at 3500 rpm for 15 min. 490 g of base grease and 10 g of graphene was divided into five parts (100 g of base grease, 99 g of base grease with 1 g of graphene, 98 g of base grease with 2 g of graphene, 97 g of base grease with 3 g of graphene, 96 g of base grease with 4 g of graphene). Owing to the “shear-thinning” phenomena, the viscosity of the base grease decreased a lot at the high shear rate (3500 rpm) and the graphene could be dispersed in grease effectively at this frequency. Moreover, the high shear stress makes it impossible for the graphene to agglomerate together. Thus, we obtained graphene-reinforced grease with superior even dispersion.

### Characterizations

The graphene was investigated by a scanning electron microscope (SEM, Quanta FEG250, USA) operating at an accelerating voltage of 10 kV. X-ray photoelectron spectroscopy (XPS) was carried out with an AXIS Ultra DLD spectrometer (Kratos, Japan). Atomic force micrographs (AFM) were obtained with a Dimension 3000 scanning probe microscope (Veeco, USA) in tapping mode. The Raman spectra were recorded using a Reflex Raman system (Renishaw plc, Wotton-under-Edge, UK) with a laser wavelength of 532 nm. The viscosity of the graphene-reinforced grease with different graphene concentrations was determined using a rotary rheometer (Physical MCR-301, Austria) at 25 °C. The friction and anti-wear properties were performed *via* the four-ball test (MS 10-A, Xiamen Tenkey Automation Co., Ltd, China), as shown in Fig. S1.† The long-term friction and wear test during the tribology test was kept at 400 N, and the frequency was 20 Hz (the speed was 1200 rpm) for one hour at 75 °C (based on standard ASTM D2266-67 C81). The friction model is shown in Fig. S2.† The upper ball under load was rotated against three stationary lower balls and the ball contact space was full of grease. The machine was cleaned and the balls were cleaned by ultrasonication at the beginning of each test. The tests were repeated at least 4 times to confirm the reproducibility of the results. The WSD (mm) was measured by electron microscope (TDS-0745-MV, Japan) then we calculated the average WSD of each ball. The thermal conductivity of the graphene grease was measured using an LFA 467 Nano Flash (Netzsch, Germany) (based on standard ASTM E 1461). In brief, the laser source emits a light pulse on the sample surface under certain temperature conditions so that the surface absorbs light energy and the temperature is instantly raised, and heat source conduction occurs towards the cold side (the upper surface). An infrared detector continuously measures the temperature of the upper surface. The data were obtained *via* a series of calculations. The infrared thermal images were captured with a Ti400 infrared camera (Fluke, USA).

## Results and discussion

### Characterization of graphene

**Fig. 1:**
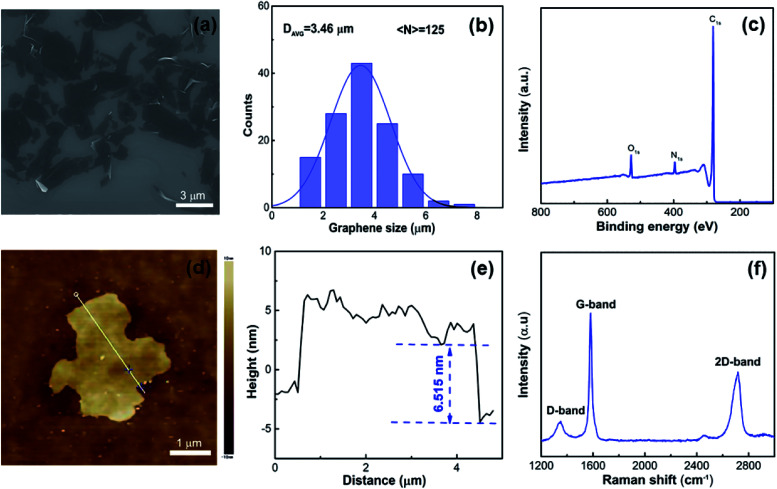
Structural characterization of graphene. (a) SEM image of graphene. (b) Particle size distribution of the graphene material according to SEM analysis. (c) XPS spectrum of graphene. (d) AFM image of graphene. (e) Cross-sectional height profile taken along the white straight line. (f) Raman spectrum of graphene.

The morphology and structure of the graphene were characterized by using SEM/AFM and XPS and Raman spectra, respectively, as shown in Fig. 1. The SEM image in Fig. 1(a) clearly shows that the size of the graphene samples was 1–8 μm. The average lateral size of graphene was determined as 3.46 μm by counting 125 pieces under our experimental conditions, as shown in Fig. 1(b). Fig. 1(c) shows the XPS survey spectrum of graphene in the range of 0–800 eV, which was used to quantify the surface element composition, namely: carbon was 93.66% and oxygen was 6.34%. Detailed analysis of the XPS spectra offers more clear evidence that the graphene is made of different functional groups and the C 1s core level spectra are illustrated in Fig. S3.† It revealed that the peaks appeared at 280.5, 281.5 and 282.5, which derived from C=C, C–C, and C–O, respectively. AFM is a valid technique for judging the thickness of graphene. The AFM image and the corresponding height profile of the graphene, from which the thickness was found to be approximately 6.5 nm, are shown in Fig. 1(d) and (e), respectively. According to the SEM and AFM images, we found that the aspect ratio, defined as the ratio of the nanosheet's length to thickness, is approximately 532. Raman spectroscopy is an effective method for studying the nanocrystalline and amorphous nature of graphene nanosheets.^28^ The D band at ∼1330 cm^−1^ and the G band at ∼1580 cm^−1^ are composed of sp^3^ hybridized carbon and sp^2^ bonded carbon atoms, respectively.^29^

In addition, the ratio of the D band and the G band (*I*_D_*/I*_G_) demonstrates the quality of the graphene nanosheets.^30^Fig. 1(f) shows the low-intensity ratio of the D band and the G band, which indicates that there are few defects in the graphene nanosheets. Few defects in the graphene means that the graphene has a comparatively complete microstructure and a smooth atomic surface, which ensures that the graphene we used exposed the tribological and thermal conductivity properties.

### Viscosity of grease

**Fig. 2:**
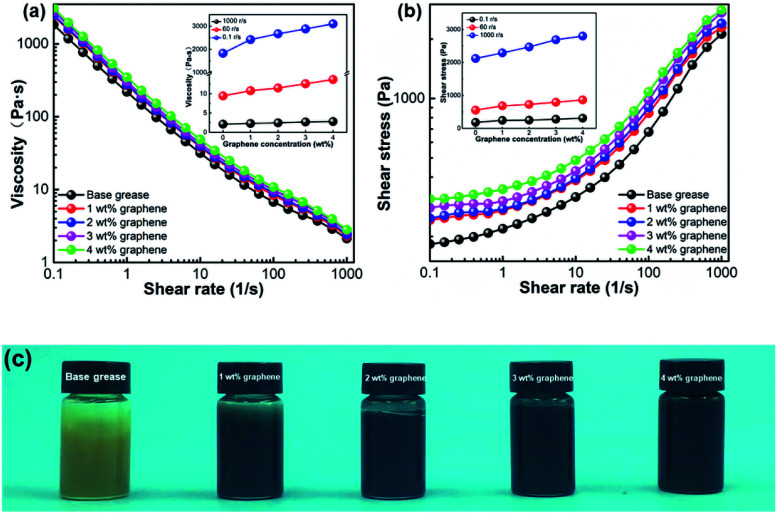
Log–log plots of (a) viscosity [the inset is (a) viscosity and (b) shear stress *versus* graphene concentration under different shear rates] and (b) shear stress *versus* shear rate for grease with different concentrations of graphene at 25 °C. (c) Pictures of grease with different concentrations of graphene.

As a non-Newtonian fluid, the viscosity of the grease will be weakened at higher shear rates, which is called “shear-thinning” phenomena.^31,32^ After adding the graphene particles, the viscosity of the grease increased a lot at lower shear rates, and changes little at higher shear rates, as shown in Fig. 2(a). With the gradual increase of the graphene content in the grease gradually, the shear stress and viscosity of the grease increased a lot. When the graphene concentration reached 4 wt%, the viscosity of the graphene grease arrived at the maximum value above these greases. Comparing the graphene grease at different shear rates (0.1, 60, and 1000 r s^−1^), the viscosity and shear stress of graphene grease changed a lot at 0.1 r s^−1^. Meanwhile, the viscosity increased slowly at the shear rate of 60 r s^−1^, which was the shear rate we used for blending the graphene grease we prepared. There was no obvious change at the rate of 1000 r s^−1^, as shown in Fig. 2(a) and (b). The base grease on its own and with different levels of graphene are shown in Fig. 2(c). We found that the graphene nanosheets were dispersed well in the base grease, which demonstrates that the prepared graphene-reinforced grease can be stored stably for a long time. Optical microscopy photos of the graphene-reinforced grease (in the concentration range from 1 to 4 wt%) are shown in Fig. S4.† These photos show the dispersion and the content of the graphene in the grease.

### Tribological properties of grease

**Fig. 3:**
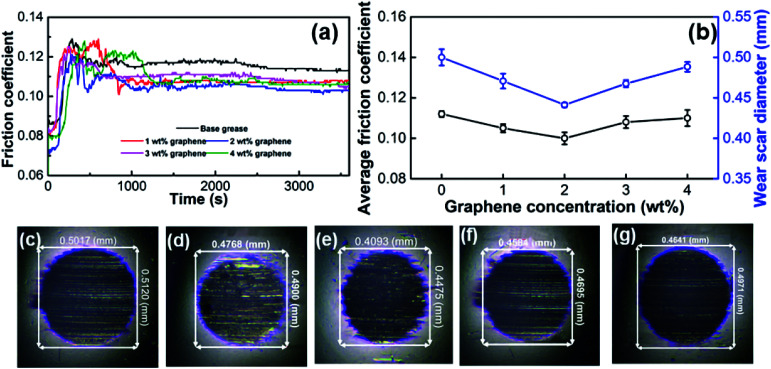
Tribological properties of graphene-reinforced grease at a frequency of 20 Hz under 400 N load at 75 °C for 1 h. (a) Friction curve. (b) Average friction coefficient and average wear scar diameter. (c)–(g) Optical microscopy images of wear scar.

**Fig. 4:**
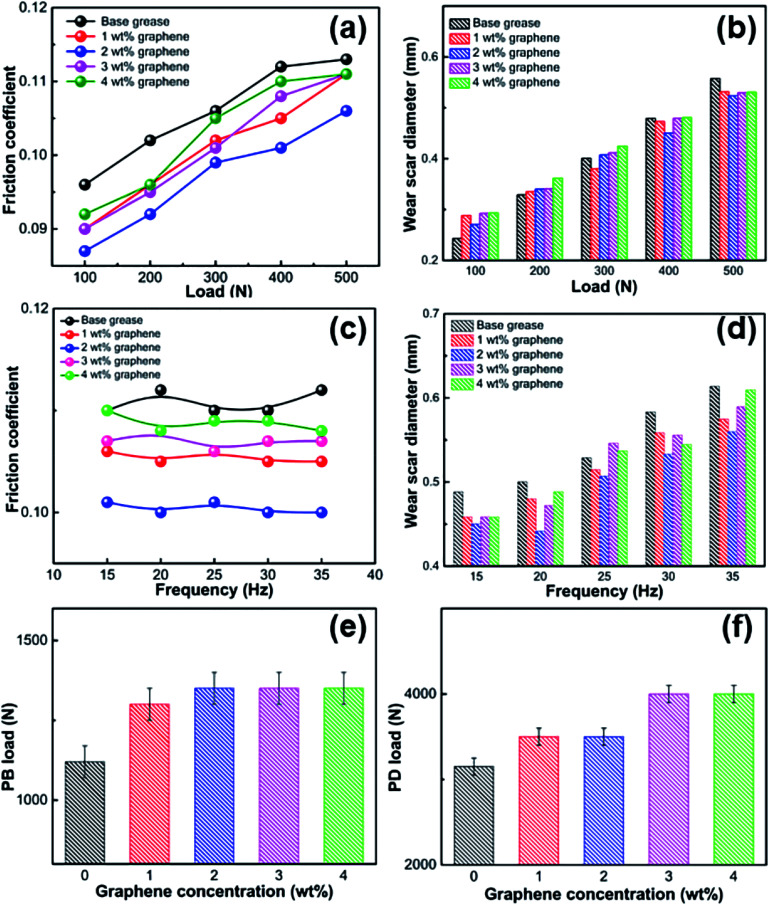
Average friction and wear scar diameters under different loads or frequencies. Average friction coefficient (a and c) and wear scar diameter (b and d) under different loads or frequencies in base grease added with different levels of graphene. (e) PB load and (f) PD load as a function of graphene concentration in base grease.

The tribological properties of the base grease and the graphene-reinforced grease, such as friction coefficient and wear, are shown in Fig. 3 and 4. In friction motion, the friction contact surface would be scratched over time through the friction of reciprocating motion with an applied load and frequency.^33^ The variation of the friction coefficient as a function of time when lubricated by base grease with different levels of graphene ranging from 0 to 4 wt% is shown in Fig. 3(a). The friction coefficients of the greases increased at first, because these greases did not participate in frictional motion surfaces effectively. When the grease and the nanosheets came into contact with the surface, the friction coefficients decreased with time from 600 to 1200 s. Finally, the friction coefficients became stable in the frictional motion surfaces. We found that the friction coefficient of the grease with 2 wt% graphene nanosheets was lowest, which showed that graphene dispersed in grease could be effective for reducing friction. The average values of the friction coefficients of these greases, from four repeated experiments, decreased with increasing graphene content until 2 wt% graphene, as shown in Fig. 3(b). It can be seen that the friction coefficient firstly decreased as the graphene concentration increased from 0 to 2 wt%, and then increased as the graphene concentration increased from 2 to 4 wt%. The statistical results suggested that the lowest average friction coefficient was 0.10 when the graphene concentration was 2 wt%. Compared with that of the base grease (0.118), the average friction coefficient of 2 wt% graphene grease decreased by 15.3%. It can be seen that the lowest friction coefficient was obtained with graphene grease with 2 wt% graphene. The typical wear scars of the balls after testing are shown in Fig. 3(c–g). Fig. 3(c–g) show the WSD of grease with 0–4 wt% graphene, respectively. These balls were the same size; a lower wear scar diameter means higher anti-wear performance. Loading with 2 wt% graphene results in the lowest wear scar diameter (0.43 mm), as shown in Fig. 3(e), which was by 14% lower than that of the base grease (0.50 mm), as shown in Fig. 3(c).

By calculating the Hertzian contact diameters,^34^ we could get the friction state. For the four-ball test with all of the balls the same size, the Hertzian formula could be simplified as follows:1
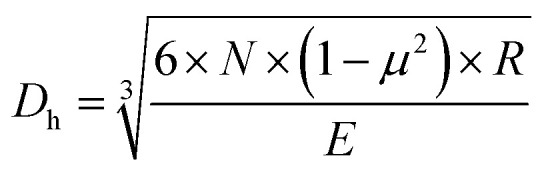
where *N* is the positive pressure on the contact friction surface under the four-ball test, *μ* is the friction factor of the ball friction surface, *R* is the radius of the balls, and *E* is 2.085 × 10^5^. The Hertzian contact diameter was calculated to be 0.30 mm under 400 N load, which is 31% lower than that of 2 wt% graphene grease, which means the lubrication state belongs to boundary lubrication. The average diameters of the wear scars were measured by the four-ball test from four repeated experiments, as shown in Fig. 3(b). It was clear that the size of the WSD decreased with increasing graphene content until 2 wt% graphene, the same as with the friction coefficient. The tribological properties of the greases with different graphene concentrations revealed that the addition of graphene could increase the lubrication and anti-wear properties of the base grease.

Fig. 4 shows the influence of the graphene concentration on the friction coefficient and WSD with increasing load and frequency. The effects of different loads (100, 200, 300, 400 and 500 N) on the lubrication and anti-wear properties of the grease with different amounts of graphene from 0 to 4 wt% are shown in Fig. 4(a) and (b). Fig. 4(a) shows the friction coefficient variation of the greases with different amounts of graphene under different loads. The friction coefficient of these graphene-reinforced greases increased a lot as the load increased because the contact and shear strength of asperities in the rubbing surface make the friction force under higher load higher than that under lower load. Fig. 4(b) shows the WSD variation of the graphene-reinforced greases under different loads. The WSD of these graphene-reinforced greases also increased with increasing load. Especially, compared with the base grease, the WSD of the graphene-reinforced greases did not decrease under 100 N load, while the WSD of graphene-reinforced greases decreased a lot under 500 N load. In general, compared with the lower load, the graphene additive dispersed in the grease played a more important role in the rubbing surface under higher load, which means that the graphene particle was easier to pay a more critical role in the friction motion surface under higher load. Fig. 4(c) and (d) show the effects of different frequencies (15, 20, 25, 30 and 35 Hz) on the lubrication and anti-wear properties of the greases with different amounts of graphene. Fig. 4(c) shows the friction coefficient variation of these greases with different amounts of graphene under different frequencies. It can be seen that the friction coefficient of these graphene-reinforced greases changed little when the frequency increased from 15 to 35 Hz. However, as shown in Fig. 4(d), the WSD of these graphene-reinforced greases increased when the frequency increased from 15 to 35 Hz. Compared with the variation of the friction coefficient, the WSD of these graphene-reinforced greases showed a distinct increase with increasing frequency. This might mainly be attributed to the contact strength having more impact on the friction coefficient than the shear strength of the asperities in the rubbing surface. In addition, we found that the portrait WSD depended on frequency and while landscape one depended on load in the four-ball test machine.

The maximum non-seizure load (PB), related to the protective film on the friction surface,^35^ of graphene-reinforced grease was investigated as shown in Fig. 4(e) (four-ball tester; speed: 1770 rpm, duration: 10 s). The PB value of base grease and grease with 1 wt% graphene is 1120 and 1300 N, respectively. The PB values of the greases with 2, 3, and 4 wt% graphene were all 1400 N, which is an improvement of 25% compared with that of the base grease. The load-carrying capacity of the grease improved with the addition of graphene. It indicated that, when the graphene is effectively dispersed in the grease, the graphene enhances the oil film strength so that the oil film cannot be broken easily. The maximum sintering load (PD) of the graphene-reinforced grease was investigated as shown in Fig. 4(f) (four-ball tester; speed: 1770 rpm, duration: 10 s). The PD value of the base grease was 3150 N. The PD values for the greases with 1 and 2 wt% graphene were both 3500 N, an improvement of 11% compared with that of the base grease. The greases with 3 and 4 wt% graphene both had PD values of 4000 N, an improvement of 27% compared to the base grease. The PD value corresponds with the complete failure value of the oil film on the friction surface. The test results showed that the PD value of grease with 4 wt% graphene increased a lot, indicating that the protective oil film on the friction surface was enhanced, which was similar to the PB of the graphene-reinforced grease.

### Thermal conductivity of grease

**Fig. 5:**
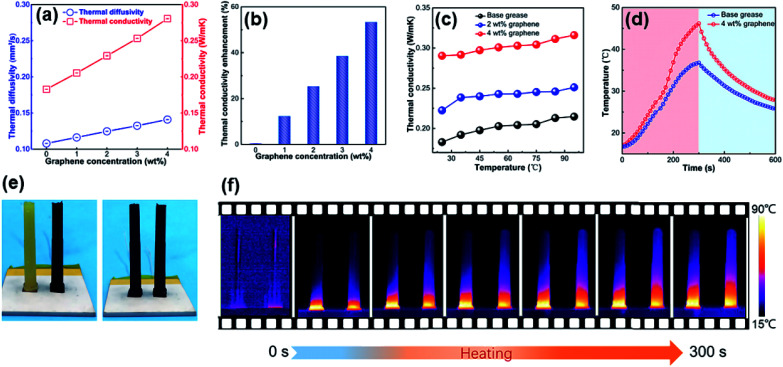
(a) Thermal conductivity and thermal diffusivity of base grease with different amounts of graphene. (b) Thermal conductivity enhancement of graphene-reinforced grease. (c) Temperature-dependent effective thermal conductivity of graphene grease. (d) Surface temperature variation of the base grease and grease with 4 wt% graphene with time upon heating and cooling events according to the infrared thermal images. (e) Photographs illustrating before and after coating with graphite. (f) Infrared thermal image variations upon heating.

Many researchers have found that the thermal conductivity of composites can be improved with the addition of graphene into the composites.^36–38^ It was found that the thermal conductivity of the graphene-reinforced greases increased with increasing graphene content. The thermal conductivity of the base grease was 0.18 W (m K)^−1^ at 25 °C. When the graphene concentration was increased to 4 wt%, the thermal conductivity increased to 0.28 W (m K)^−1^, as shown in Fig. 5(a). Compared with the base grease, the thermal conductivity of the grease with 4 wt% graphene was increased by 55.6%, as shown in Fig. 5(b). As more graphene is dispersed in the grease, the density of the heat conduction connecting channels between the graphene layers is likely to be greater, so that the thermal conductivity can be effectively improved. To test the temperature dependence of the greases with different graphene contents, we studied the thermal conductivity from 25 to 95 °C in Fig. 5(c). We found that the thermal conductivity increased a little with increasing temperature. The thermal diffusivity of the graphene-reinforced grease decreased, but the specific heat capacity increased. Owing to the low level of graphene dispersed in the grease, the temperature dependence is similar for all of the graphene-reinforced greases. For the purpose of demonstrating the thermal conductivity of the graphene-reinforced grease, we studied the surface temperature variation of the base grease and grease with 4 wt% graphene with time upon heating and cooling according to the infrared thermal images in Fig. 5(d). As shown in Fig. 5(e), we designed a simple container to hold the graphene grease for comparing the temperature changes of base grease and grease with 4 wt% graphene. To accelerate the heat transfer and ensure a uniform heating process, we placed the samples on a piece of copper foil. To ensure that the samples could stand firmly on the table, we used glue to connect the copper foil and the samples. Both samples were coated with graphite and heated by using a heating station (a resistor turns on the current and then produces a constant heat) from room temperature to 100 °C. An infrared camera was used to record the surface temperature variation with heating time. Fig. 5(f) presents the infrared images recorded from 0 to 300 s. We compared representative images to confirm that the thermal conductivity of the grease with 4 wt% graphene is higher than that of the base grease. The lubrication and heat dissipation model for graphene-reinforced grease during friction is proposed, as shown in Fig. S5.† During friction, the grease with graphene was proposed to have a lower friction coefficient and wear rate. Besides, less friction heat will be generated upon the friction surface owing to the low friction coefficient and wear. However, the friction heat is more easily diffused with the addition of graphene in the grease, which is attributed to the formation of the thermal pathway between graphene nanosheets. The tribological and thermal conductive properties of the base grease are improved simultaneously when graphene is dispersed in the grease.

## Conclusions

In summary, the tribological behavior and thermal conductivity of base grease reinforced with graphene were studied. The lubrication and anti-wear properties of the base grease were ameliorated with the addition of graphene, indicating that these graphene-reinforced greases show better tribological properties than those of the base grease. Compared with the base grease, the friction coefficient and anti-wear properties of the graphene-reinforced grease were reduced by 15.3% and 14% under 400 N load and 20 Hz frequency, respectively. We investigated the effects of load and frequency on the lubrication and anti-wear properties of the greases with different concentrations of graphene and found that the friction coefficient increased with increasing load but not with increasing frequency. Meanwhile, the thermal conductivity of the graphene-reinforced grease increased with increasing graphene concentration. The thermal conductivity of the grease with 4 wt% graphene was 0.28 W (m K)^−1^ at 25 °C, which is an increase of 55.6% compared with the base grease. It was proposed that the addition of graphene into base grease could not only enhance the tribological properties but also increase the thermal transportation performance.

## Conflicts of interest

There are no conflicts to declare.

## Supplementary Material

RA-009-C9RA09201C-s001
