# Supplementary material for: Graphene as a nanofiller for enhancing the tribological properties and thermal conductivity of base grease
Source: RSC Adv. 2019 Dec 20;9(72):42481–8. doi: 10.1039/c9ra09201c (PMC9076656; doi:10.1039/c9ra09201c)
Supplement: RA-009-C9RA09201C-s001 [file RA-009-C9RA09201C-s001.pdf]

Electronic Supplementary Material (ESI) for RSC Advances.
This journal is © The Royal Society of Chemistry 2019

SUPPORTING INFORMATION

Graphene as Nanofiller for Enhancing Tribological Property and Thermal Conductivity of Base Grease

Hui Fu, [a,b] *Guoping Yan,* [a,*] *Meng Li,* [b] *Hao Wang,* [b] *Yapeng Chen,* [b,c], *Chao Yan,* [d] *Cheng-Te Lin,* [b,c] *Nan Jiang,* [b,c*] *Jinhong Yu* [b,c *]

[a] *College of Materials Science and Engineering, Wuhan Institute of Technology, Wuhan 430073, Hubei, China.*

[b] *Key Laboratory of Marine Materials and Related Technologies, Zhejiang Key Laboratory of Marine Materials and Protective Technologies, Ningbo Institute of Materials Technology and Engineering, Chinese Academy of Sciences, Ningbo, 315201, China.*

[c] *Center of Materials Science and Optoelectronics Engineering, University of Chinese Academy of Sciences, Beijing 100049, China.*

[d] *School of Materials Science and Engineering, Jiangsu University of Science and Technology, Zhenjiang, 212003, China.*

Corresponding author, E-mail: guopyan2006@163.com; jiangnan@nimte.ac.cn; yujinhong@nimte.ac.cn.

SUPPLEMENT

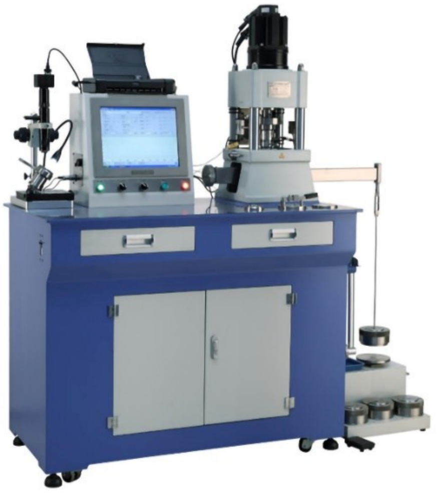

Figure S1 Digital photographs of the four-ball testing machine.

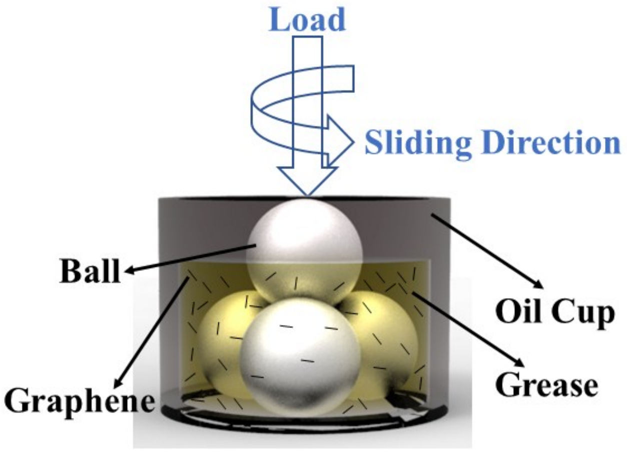

Figure S2 Schematic illustration of four-ball testing model.

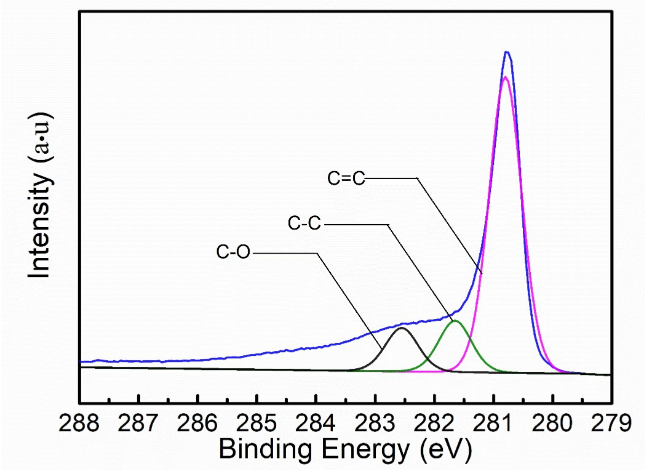

Figure S3 Corresponding C1s XPS spectra of graphene.

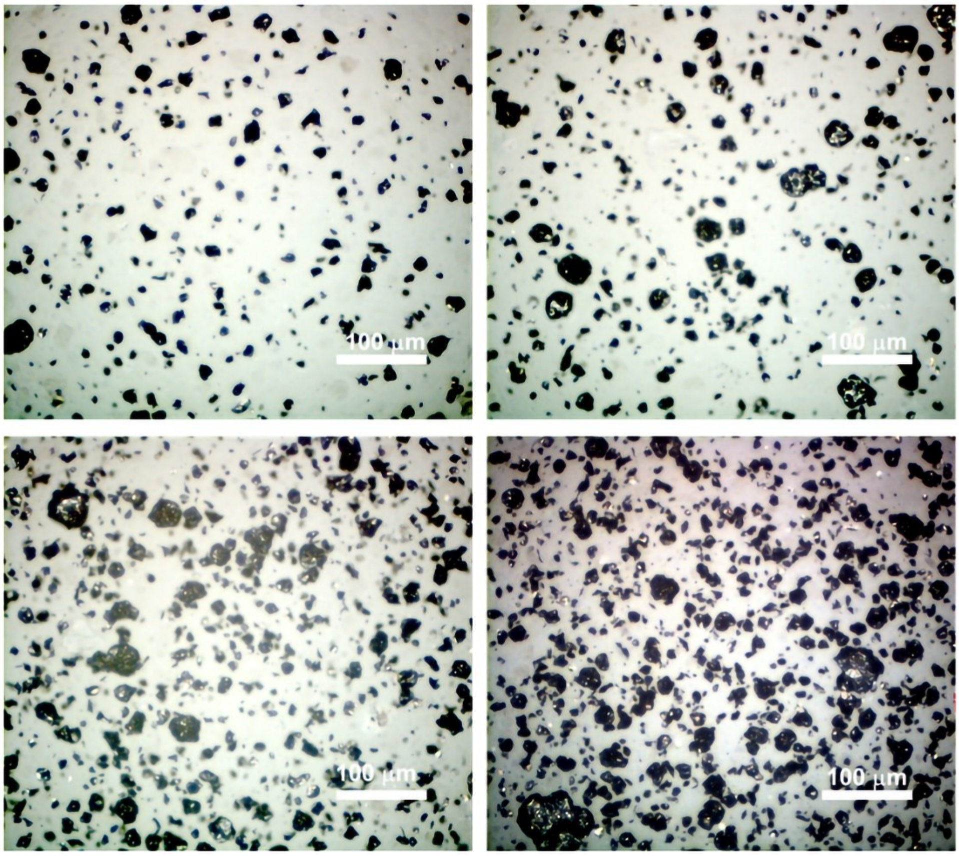

Figure S4 Optical microscope photos of these graphene.

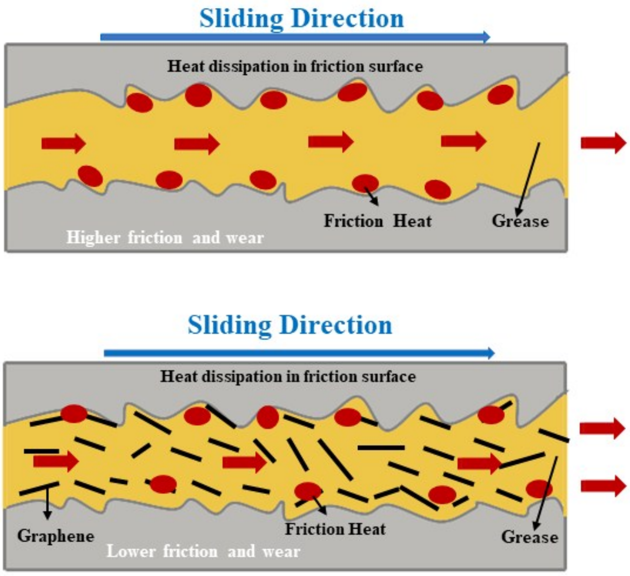

Figure S5 Lubrication and heat dissipation model of graphene grease.
